# Conserved and Specific Root-Associated Microbiome Reveals Close Correlation Between Fungal Community and Growth Traits of Multiple Chinese Fir Genotypes

**DOI:** 10.3390/microorganisms13081741

**Published:** 2025-07-25

**Authors:** Xuan Chen, Zhanling Wang, Wenjun Du, Junhao Zhang, Yuxin Liu, Liang Hong, Qingao Wang, Chuifan Zhou, Pengfei Wu, Xiangqing Ma, Kai Wang

**Affiliations:** 1 College of Forestry, Fujian Agriculture and Forestry University, Fuzhou 350002, China; 2 Chinese Fir Engineering Research Center of National Forestry and Grassland Administration, Fuzhou 350002, China; 3 Co-Innovation Center for Sustainable Forestry in Southern China of Jiangsu Province, Key Laboratory of Soil and Water Conservation and Ecological Restoration of Jiangsu Province, Nanjing Forestry University, Nanjing 210037, China

**Keywords:** Chinese fir, rhizosphere soil, root endophyte, microbiome, trade-off

## Abstract

Plant microbiomes are vital for the growth and health of their host. Tree-associated microbiomes are shaped by multiple factors, of which the host is one of the key determinants. Whether different host genotypes affect the structure and diversity of the tissue-associated microbiome and how specific taxa enriched in different tree tissues are not yet well illustrated. Chinese fir (*Cunninghamia lanceolata*) is an important tree species for both economy and ecosystem in the subtropical regions of Asia. In this study, we investigated the tissue-specific fungal community structure and diversity of nine different Chinese fir genotypes (39 years) grown in the same field. With non-metric multidimensional scaling (NMDS) analysis, we revealed the divergence of the fungal community from rhizosphere soil (RS), fine roots (FRs), and thick roots (TRs). Through analysis with α-diversity metrics (Chao1, Shannon, Pielou, ACE, Good‘s coverage, PD-tree, Simpson, Sob), we confirmed the significant difference of the fungal community in RS, FR, and TR samples. Yet, the overall fungal community difference was not observed among nine genotypes for the same tissues (RS, FR, TR). The most abundant fungal genera were *Russula* in RS, *Scytinostroma* in FR, and *Subulicystidium* in TR. Functional prediction with FUNGuild analysis suggested that ectomycorrhizal fungi were commonly enriched in rhizosphere soil, while saprotroph–parasite and potentially pathogenic fungi were more abundant in root samples. Specifically, genotype N104 holds less ectomycorrhizal and pathogenic fungi in all tissues (RS, FR, TR) compared to other genotypes. Additionally, significant correlations of several endophytic fungal taxa (*Scytinostroma*, *Neonothopanus*, *Lachnum*) with the growth traits (tree height, diameter, stand volume) were observed. This addresses that the interaction between tree roots and the fungal community is a reflection of tree growth, supporting the “trade-off” hypothesis between growth and defense in forest trees. In summary, we revealed tissue-specific, as well as host genotype-specific and genotype-common characters of the structure and functions of their fungal communities.

## 1. Introduction

Forests represent one of the most complex ecological systems on Earth, covering more than 40 million square kilometers and accounting for approximately 30% of the planet’s terrestrial surface. Forest ecosystems are distributed across most of the world’s biomes and significantly contribute to global biodiversity [1]. Historically, forests have been a major focus in ecological research. With the advancement of research, our understanding of forest ecosystems has progressively evolved from a macroscopic to a microscopic perspective, shifting the emphasis from vegetation structure and community succession to the presence of microorganisms within ecosystems and the intricate and finely tuned interactions between these microbes and trees. Previous research on forest ecosystems has focused on aboveground components, including tree species composition, biodiversity, and carbon sequestration. However, growing evidence underscores the pivotal role of complex interactions between soil microbial communities and trees in shaping ecosystem stability, productivity, and resilience to environmental change.

Among belowground components, root-associated microbial communities have attracted considerable attention, serving as a vital interface between plants and the soil environment. The root microbiome is shaped through complex interactions regulated by root exudates and the plant immune system. In turn, microbial communities enhance plant adaptability and metabolic capacity, contributing to processes such as development, nutrient uptake, and stress responses [2]. Microorganisms facilitate plant growth by improving the acquisition of essential nutrients such as iron, nitrogen, and phosphorus [3,4,5], and by increasing plant resilience to abiotic stresses including drought and salinity [6,7]. Additionally, microbes can regulate root development through effects on plant gene expression [8] and modulate the host immune system to establish colonization while protecting against pathogenic invasions [9]. Therefore, root-associated microbiota not only serve as crucial mediators in plant–soil interactions but also play essential regulatory roles in root development and function.

Numerous studies have demonstrated the high diversity of root microbial communities. This diversity stems from several factors. Fungal communities, in particular, display strong host specificity, with host genotype recognized as a primary determinant shaping the structure of the root microbiome [10]. Meanwhile, bacterial communities are notably dynamic and responsive to environmental conditions. The composition of the root microbiome is largely driven by host-mediated recruitment and selection, often through the secretion of root exudates and specific signaling compounds [2]. This recruitment is environmentally sensitive; for instance, light conditions can influence the metabolic output of exudates, enriching for more metabolically active rhizosphere communities [11]. Consequently, studies on rhizosphere microbial community structures are not only vital for understanding plant survival strategies but also offer valuable insights into plant responses to environmental change.

Chinese fir (*Cunninghamia lanceolata*) is widely cultivated in East Asia due to its high economic value. However, its growth and health are increasingly challenged by soil nutrient limitations and climate change [12]. Investigating the root-associated microbial communities of Chinese fir can provide novel perspectives for sustainable plantation management. Recent research has made significant progress in characterizing the microbiota of different root compartments (rhizosphere soil, non-rhizosphere soil, fine roots, thick roots, primary roots) and has identified beneficial microbial strains [13]. Another study also isolated a strain of *Burkholderia ubonensis* from the forest soil of a Chinese fir plantation and has demonstrated strong phosphate-solubilizing capabilities and multiple growth-promoting traits, including IAA production, ACC deaminase activity, and siderophore secretion [14]. Advances have also been made in fungal research. Studies comparing fungal diversity across nine Chinese fir plantations have identified environmental factors influencing community composition [15]. Additionally, the use of beneficial fungi such as arbuscular mycorrhizal fungi has been shown to enhance phosphorus uptake and maintain root health under low-phosphorus conditions [16,17].

Despite these advances, most existing studies have focused on microbial diversity, community dynamics, and the functional roles of beneficial strains. However, as mentioned earlier, root-associated microbial communities, particularly fungi, exhibit strong host specificity, and host genotype is presumed to be a critical factor influencing fungal community structure and function. Yet, comprehensive empirical evidence to support this is still lacking. Moreover, previous studies have largely emphasized species abundance and compositional changes, with limited focus on the hierarchical structure of root-associated microbial communities. In light of this, the present study investigates nine Chinese fir genotypes grown under identical environmental conditions. Using high-throughput amplicon sequencing, we systematically analyze fungal communities in rhizosphere soil, fine roots, and thick roots. By integrating analytical approaches such as non-metric multidimensional scaling (NMDS), α-diversity indices, and FUNGuild-based functional predictions, this study aims to evaluate inter-genotype differences in rhizosphere fungal communities and constructs a hierarchical community structure across the “thick root–fine root–rhizosphere” continuum. These findings will enhance our understanding of the ecological functions of root-associated fungi in root development and provide theoretical foundations for precision cultivation and sustainable management of Chinese fir plantations.

## 2. Material and Methods

### 2.1. Sample Collection and Treatment

Samples of rhizosphere soil, fine roots, and thick roots were collected from 39-year-old Chinese fir trees representing nine distinct genotypes at Zhangping Wuyi National Forest (Zhangping, Fujian, China). These nine genotypes originated from major Chinese fir production regions across China: N94 (Hubei Province-1), N95 (Guizhou Province), N96 (Zhejiang Province), N97 (Sichuan Province), N98 (Guangdong Province), N100 (Guangxi Province), N102 (Jiangxi Province), N103 (Hubei Province-2), and N104 (Fujian Province). All genotypes were cultivated under the same environmental conditions at the Zhangping site to minimize the influence of site-related variability and to isolate the effect of genotype. Sample collection was conducted in the morning (9:00–12:00 a.m., ambient temperature 30–33 °C) in July. For each individual tree, three sampling points around the trunk (approximately equidistant and evenly distributed to reduce microenvironmental heterogeneity) were selected, and samples from the three points were pooled to form one biological replicate. For each genotype, three individual trees were selected, yielding three biological replicates per genotype. All samples were collected at a depth of 20–40 cm from the ground surface. After collection, roots with rhizosphere soil were kept in cool boxes with frozen ice bags and processed within 48 h to preserve microbial integrity. Rhizosphere soil was washed from fine roots with sterile water by centrifuging at 4000 rpm for 5 min in 50 mL tubes. Fine roots (diameter < 2 mm) and thick roots (diameter > 2 mm) were further washed with 0.025% Silwet solution at 80 rpm for 1 h, followed by a 1 min wash in 75% ethanol [12,13].

### 2.2. DNA Extraction and ITS Amplicon Sequencing

Genomic DNA in soil and root samples was extracted using the HiPure Soil DNA kit (Magen, Guangzhou, China) and HiPure Stool DNA kit (Magen, Guangzhou, China). For fungal community profiling, the internal transcribed spacer 1 (ITS1) region of fungal ribosomal DNA was amplified using primers ITS1-F (CTTGGTCATTTAGAGGAAGTAA) and ITS2 (GCTGCGTTCTTCATCGATGC). The PCR amplification protocol involved an initial denaturation at 95 °C for 5 min, followed by 30 amplification cycles consisting of denaturation at 95 °C for 1 min, annealing at 50 °C for 1 min, and extension at 72 °C for 1 min, with a final elongation at 72 °C for 7 min. Amplified ITS1 fragments were visualized and isolated using 2% agarose gel electrophoresis and subsequently purified with a commercial gel extraction kit (Axygen Biosciences, Union City, CA, USA). DNA concentrations were measured using a Qubit 3.0 fluorometer (Thermo Fisher Scientific, Waltham, MA, USA), and quality was verified using a real-time PCR instrument (Life Technologies, Foster City, CA, USA). All PCR products were normalized to the same molar concentration and pooled for high-throughput sequencing using the Illumina NovaSeq 6000 platform (San Diego, CA, USA) with 250 bp paired-end reads (PE250). The resulting raw sequencing reads were deposited into the NCBI Sequence Read Archive (SRA) under accession number PRJNA1268739.

### 2.3. Amplicon Bioinformatics Analysis

Raw ITS reads were initially processed using FASTP (version 0.18.0) [18] to eliminate low-quality sequences and residual adapter contamination. Specifically, sequences were discarded if they contained over 10% ambiguous nucleotides (N) or if fewer than half of the bases had a quality score above Q20. Clean paired-end reads were subsequently merged using FLASH (version 1.2.11), requiring a minimum overlap of 10 base pairs and allowing for a mismatch rate of no more than 2% [19]. The resulting merged sequences, referred to as raw tags, were then filtered to remove spurious reads based on predefined noise thresholds [20], yielding high-confidence clean tags for downstream analysis. Operational Taxonomic Units (OTUs) were clustered using the UPARSE pipeline (version 9.2.64) [21] with a similarity threshold of 97%. During this step, chimeric sequences were identified and removed via UCHIME to ensure only genuine fungal sequences were retained [22]. The most abundant sequence in each OTU cluster was selected as the representative sequence. Taxonomic classification of these representative sequences was conducted using the RDP Classifier with a confidence cutoff of 0.8, referencing the UNITE fungal ITS database. Taxonomic composition results were visualized using Krona v2.6 [23], and genus-level community profiles were illustrated with the ggplot2 package v2.2 [24] in R.

To ensure unbiased diversity comparison across samples, rarefaction was applied to standardize sequencing depth. Alpha diversity metrics, including Chao1, ACE, Shannon, Simpson, Pielou’s evenness, and Faith’s PD, were calculated using QIIME [25]. Differences in alpha diversity indices among sampling compartments and genotypes were statistically tested using Tukey’s HSD test within the Vegan package (version 2.5.3) [26] in R. Beta diversity was evaluated through non-metric multidimensional scaling (NMDS) based on Bray–Curtis dissimilarity, also implemented via the Vegan package [26]. Sample clustering patterns were used to assess variation in community structure between rhizosphere soil, fine roots, and thick roots across nine different genotypes. Functional annotation of fungal communities was conducted using FUNGuild [27], assigning OTUs into function modes such as Ectomycorrhizal, Saprotroph–Parasite, Plant Pathogen, and Unassigned/Others, and results were visualized by genotype with ggplot2 [24]. Pearson correlation analysis between tree growth indexes (tree height, diameter at breast height, and relative stand volume) and species abundance was performed using the psych package (version 2.4.6) in R 4.2.3 [28].

## 3. Results

### 3.1. Divergence of the Fungal Community from Rhizosphere Soil, Fine Roots, and Thick Roots

**Figure 1:**
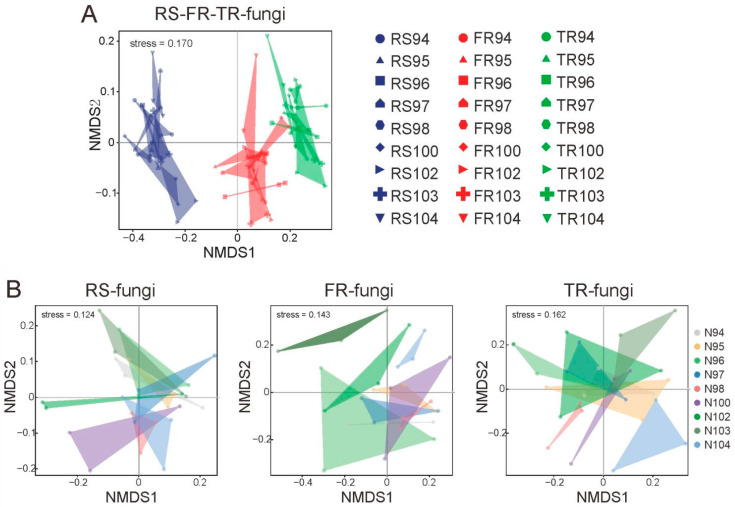
NMDS analysis of fungal communities from different genotypes and plant compartments. (**A**) NMDS (non-metric multidimensional scaling) analysis of fungal communities in three compartments: RS (rhizosphere soil), FR (fine root), and TR (thick root). Different colors represent different compartments, and different shapes indicate sampling genotypes (N94–N104). NMDS stress = 0.170. (**B**) NMDS analyses of fungal communities within each compartment (RS, FR, TR), showing differences among genotypes (N94-N104). RS NMDS stress = 0.124. FR NMDS stress = 0.143. TR NMDS stress = 0.162.

To elucidate the diversity and structural dynamics of root-associated fungal microbiota across varying tissue types and genetic backgrounds, we analyzed fungal communities derived from the thick roots, fine roots, and rhizosphere soil of Chinese fir trees of nine distinct genotypes. Non-metric multidimensional scaling (NMDS) analysis of fungal beta-diversity (STRESS = 0.170) (Figure 1A) revealed a key insight: the genotype of *C. lanceolata* exerted a comparatively weaker influence on the beta-diversity of fungal communities than tissue type, underscoring tissue specificity as a predominant determinant of microbiome structure. Notably, the compositional divergence between fungal communities in fine roots and rhizosphere soil was relatively modest, suggesting potential fungal migration from the rhizosphere into the fine root compartment.

In addition to exploring the heterogeneity of fungal microbiomes between different tissues, the diversity of microbiomes within the same tissue is also well worth attention. We can observe that the analyzed samples show an overall tendency to cluster, indicating that the root microbiome exhibits a certain degree of stability (Figure 1B). However, tissue-specific distribution patterns were evident: fungal diversity followed a gradient, increasing from thick roots to fine roots and peaking in rhizosphere soil. Rhizosphere soil (RS) samples exhibited broader dispersion, reflecting elevated community heterogeneity likely driven by variable environmental inputs and diverse root exudates. Fine root (FR) samples clustered between those of RS and thick roots (TRs), consistent with their role as a transitional interface in plant–soil–microbe interactions. This intermediate clustering suggests that while FR fungal communities may partially originate from rhizosphere colonization, they are likely shaped by selective pressures imposed by the host. In contrast, TR samples displayed tight clustering with low intra-group variation, indicative of higher compositional stability and potential specialization of fungal taxa in thick root tissues, likely attributable to stronger host-mediated selection and compartmentalization. Collectively, these patterns illustrate a gradient from open (soil-influenced) to closed (host-controlled) microbial communities, providing preliminary evidence for the dynamic processes of fungal migration, colonization, and stabilization along the soil–root continuum. Interspecific comparisons further revealed significant variations in fungal diversity across genotypes: N98 and N94 genotypes demonstrated relatively stable communities, whereas N100 and N104 exhibited higher diversity.

### 3.2. The α-Diversity of the Fungal Community from Different Tissues

**Figure 2:**
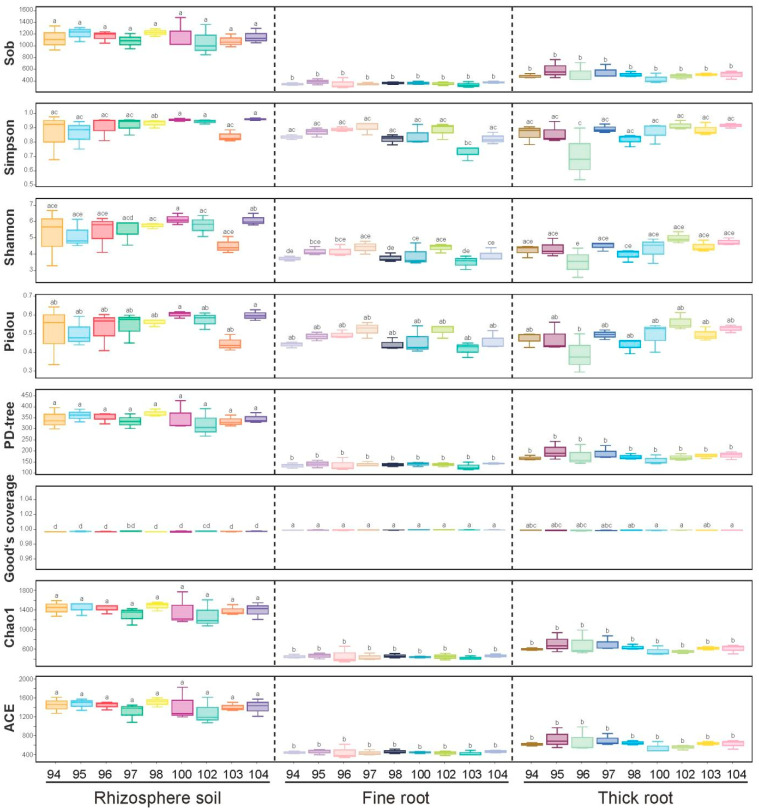
Fungal community α-diversity indices of different genotypes (N94-N104) across three compartments. Tukey’s Honestly Significant Difference (HSD) test was implemented to calculate the difference among indices. Different letters on top of bars indicate significant differences.

Alpha diversity analysis revealed a pronounced spatial gradient in fungal richness and evenness across different tissue types. Analyses based on Sobs, Chao1, and ACE indices showed that rhizosphere soil (RS) samples from nine Chinese fir genotypes exhibited significantly higher species richness compared to fine roots (FRs) and thick roots (TRs) (Figure 2). The Shannon and Simpson indices further illustrated the variation trends in fungal community diversity and evenness, with both metrics showing significantly higher values in rhizosphere soil (RS) compared to fine roots (FRs) and thick roots (TRs). These results indicated that the rhizosphere fungal community was not only richer in species but also more evenly structured. In contrast, fungal communities within root tissues were typically dominated by a few highly abundant taxa, resulting in lower overall evenness and suggesting a selective filtering effect by the host root environment.

In rhizosphere soil (RS), genotypes N100, N102, and N104 exhibited the highest Simpson indices, suggesting a more balanced and diverse fungal community structure. This pattern may reflect an enhanced capacity for microbial homeostasis and ecological adaptability in the rhizosphere of these genotypes. In addition, analysis of the phylogenetic diversity (PD-tree) index showed that the rhizosphere soil (RS) samples exhibited significantly higher values than fine roots (FRs) and thick roots (TRs) at the phylogenetic lineage level. This indicated that RS harbored a broader spectrum of evolutionary taxa, further reinforcing its role as a biodiversity “hotspot” and a potential reservoir of functional microbial traits. The Good’s coverage index was close to or exceeded 0.98 across all samples, suggesting sufficient sequencing depth and high data completeness, thereby ensuring that the fungal community structures obtained were highly representative and reliable for comparative analysis.

### 3.3. Genus-Level Fungal Community Structure Analysis

**Figure 3:**
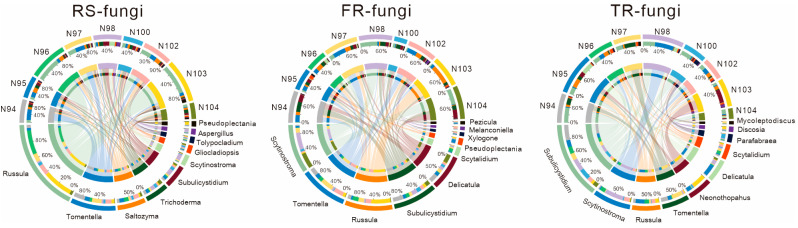
Genus-level composition of fungal communities from different genotypes (N94–N104) across three compartments. RS-fungi: fungal genera in rhizosphere soil; FR-fungi: fungal genera in fine roots; TR-fungi: fungal genera in thick roots.

The chord diagram illustrated the associations and relative abundance distributions of fungal genera across different tissues of Chinese fir (rhizosphere soil, fine roots, thick roots) and various genotypes (N94-N104) (Figure 3). The analysis revealed that fungal community compositions exhibited distinct spatial distribution patterns among RS, FRs, and TRs. Moreover, the dominant genera showed significant variation across both tissue types and genotypes.

In the rhizosphere soil, *Russula* and *Tomentella* were identified as the dominant fungal genera. Specifically, *Russula* was significantly enriched in the N96 and N103 genotypes, while *Tomentella* was predominantly enriched in N94 and N97. The consistently high abundance of *Russula* and *Tomentella* across multiple genotypes reflects their strong ecological adaptability and wide distribution, suggesting that they may play an important symbiotic role in supporting nutrient uptake and stress resistance in Chinese fir. Additionally, although *Saltozyma*, *Trichoderma*, and *Subulicystidium* were not dominant, their stable presence across all genotypes indicates a potential broad-spectrum adaptability, implying that they may serve as key contributors in maintaining the rhizosphere ecosystem. In fine roots, *Scytinostroma* and *Tomentella* were identified as the dominant genera, reflecting a selective preference of plant root tissues for specific symbiotic fungi. *Scytinostroma* was significantly enriched in genotypes N98 and N104, while *Tomentella* was predominant in N97 and N102. In N103, *Russula* was the dominant genus, accompanied by a reduced presence and lower diversity of other fungal taxa. In addition, *Delicatula* exhibited relatively high abundance in N104 and N94. Notably, *Xylogone* and *Melanconiella* were exclusively detected in fine root samples, suggesting their potential role as tissue-specific endophytic fungi. In thick roots, *Subulicystidium* and *Scytinostroma* were identified as the dominant fungal genera. *Subulicystidium* was distributed across nearly all genotypes and showed relatively high abundance in N94, N98, and N100, while *Scytinostroma* was significantly enriched in N98. Other genera, including *Russula*, *Tomentella*, *Neonothopahus*, and *Delicatula*, also exhibited prominence in specific genotypes. For example, *Neonothopahus* dominated the fungal community in N103, while *Delicatula* was significantly enriched in N95, followed by *Russula* and *Subulicystidium*. *Tomentella* was predominant in N97, and *Subulicystidium* remained the most abundant genus in N100.

By comparing rhizosphere soil samples with root tissue samples, we found that the majority of fungal taxa present in root tissues were also detected in the rhizosphere. For instance, dominant genera such as *Scytinostroma*, *Tomentella*, *Russula*, and *Subulicystidium*, which were abundant in root tissues, were also widely distributed in the rhizosphere soil. These results suggested that the fungal communities colonizing Chinese fir roots were largely derived from the surrounding rhizosphere, indicating that rhizosphere soil may serve as a major reservoir for root-colonizing fungi. Furthermore, root exudates and tissue-specific characteristics likely played a selective and enriching role during the colonization process, determining which fungal taxa successfully migrated from soil to root compartments and established stable associations.

Meanwhile, certain fungal genera exhibited pronounced tissue-specific distribution patterns. For example, *Delicatula* and *Scytalidium* were exclusively detected in root tissues (both fine roots [FRs] and thick roots [TRs]), while *Xylogone*, *Melanconiella*, and *Pezicula* were restricted to fine roots. In contrast, *Neonothopahus* and *Parafabraea* were specifically found in thick roots. *Pseudoplectania* was predominantly present in the rhizosphere soil and fine roots but was rarely detected in thick root samples.

In addition, the genotype-specific enrichment patterns of fungal taxa further underscored the critical role of host genetic background in shaping the assembly of the rhizosphere microbiome. Certain genotypes, such as N103 and N98, consistently exhibited significant enrichment of specific dominant fungal genera across different plant tissues, suggesting that variations in root exudate composition, immune regulation mechanisms, and tissue structures among genotypes collectively influenced fungal colonization and coexistence. These spatial and genotype-associated community assembly features revealed the complexity of plant–microbe interactions and provided a theoretical foundation for precision microbiome management and microbiome-assisted breeding, ultimately contributing to improved forest health and ecosystem stability.

### 3.4. Functional Prediction Reveals Tissue-Specific and Genotype-Specific Functions

**Figure 4:**
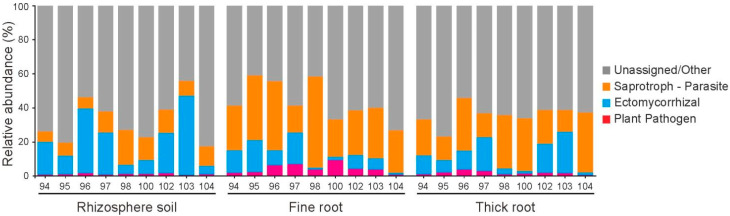
Relative abundance of fungal functional groups across three compartments for different genotypes (N94-N104). FUNGuild analysis was applied to predict the relative abundance of the fungal functions related to saprotroph–parasite, ectomycorrhizal, and plant pathogens.

The relative abundances of different functional fungal groups in various tissue parts across multiple Chinese fir genotypes were presented (Figure 4). Significant differences in the relative abundances of functional fungi were observed among tissues. Ectomycorrhizal fungi showed higher proportions in rhizosphere soil, indicating that the rhizosphere microenvironment facilitates their colonization and proliferation. The proportions of saprotroph–parasite fungi increased in fine and thick roots, suggesting that the internal root environment is more suitable for the survival of fungi with both saprophytic and parasitic characteristics. Plant pathogens generally exhibited low abundances across tissues, but slight enrichments were observed in fine and thick roots of specific genotypes, such as genotypes N96, N97, and N100. Unassigned or other fungal groups accounted for the largest proportion in all tissues and genotypes, reflecting the current gaps in our understanding of Chinese fir-root-associated fungi.

Fluctuations in the abundances of fungal groups were observed among different genotypes within the same tissue. Notably, genotype N104 showed lower abundances of both ectomycorrhizal fungi and plant pathogens across all tissues compared to other genotypes. This may indicate unique differences in the symbiotic relationship with ectomycorrhizal fungi and the resistance against plant pathogen infection in genotype N104 compared to other Chinese fir genotypes.

### 3.5. Correlation of Fungal Taxonomic Abundance with Chinese Fir Growth

**Figure 5:**
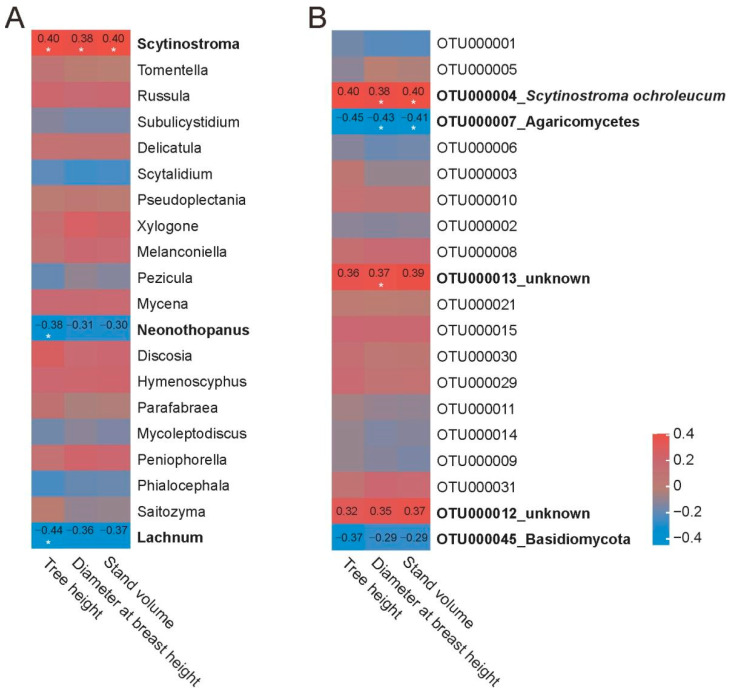
Pearson correlation analysis between Chinese fir growth indexes and fungal taxonomic abundance in fine roots. Tree height, diameter at breast height, and the relative stand volume of nine different genotypes of Chinese fir were considered as tree growth indexes. Fungal taxa in (**A**) genera and (**B**) OTU levels were selected. Correlation coefficient is indicated in heatmap when |r| > 0.3. *: *p* < 0.05.

To reveal whether specific taxonomic fungi are connected to the growth traits of different Chinese fir genotypes, we performed correlation analysis between tree growth indexes (tree height, diameter at breast height, relative stand volume) and fungal abundance. Interestingly, a significantly positive correlation between the genus *Scytinostroma* and tree growth indexes (r = 0.40, 0.38, 0.40) was observed. The genera *Neonothopanus* and *Lachnum* were negatively correlated to tree height (r = −0.38, −0.44) (Figure 5A). At the OTU level, OTU000004 (*Scytinostroma ochroleucum*) and OTU000013 were positively correlated to tree diameter at breast height and strand volume (r = 0.38, 0.40, 0.37), while OTU000007 (Agaricomycetes) had a negative correlation to tree diameter and strand volume (r = −0.43, −0.41) (Figure 5B). Additionally, there were uncharacterized OTUs that might have weak correlation (|r| < 0.3) to tree growth indexes, indicating the complexity of fungal taxa that may affect the growth traits among different genotypes of Chinese fir.

## 4. Discussion

### 4.1. The Decreasing Trend of Fungal Community from the Rhizosphere to the Root Interior

This study demonstrates that fungal diversity in the RS of Chinese fir, as assessed by species richness, community evenness, and phylogenetic diversity, is significantly higher than that in FRs and TRs. This pattern reveals a clear spatial gradient of decreasing fungal diversity from the rhizosphere to internal root compartments, highlighting the root system’s pivotal role as a selective barrier in shaping microbial colonization. The progressive decline in diversity suggests a stepwise filtering mechanism, whereby the plant increasingly restricts microbial entry and establishment as fungi transition from external soil to internal tissues [29]. The rhizosphere soil is the direct interface between plant roots and the external environment. It is the most frequent and active zone for interactions between plants and soil microorganisms. Plant roots secrete carbon sources, organic acids, and various secondary metabolites, collectively known as root exudates [30]. These exudates provide energy and nutrients to soil microbes [31]. They also significantly influence the structure and functional composition of microbial communities. Due to its high openness and dynamic nature [32], the rhizosphere is commonly regarded as a hotspot for fungal diversity [33].

In comparison, inner root tissues, particularly thick roots, constitute a more structurally enclosed and immunologically fortified microenvironment with greater internal stability [34]. Microbial colonization within these compartments is governed by a highly selective host filtering system that integrates physical barriers (e.g., cell walls), chemical defenses (e.g., antimicrobial compounds such as flavonoids and phenolic acids), and immune recognition mechanisms mediated by pattern recognition receptors (PRRs) [35]. Under this multilayered surveillance, only fungal taxa possessing specific adaptive traits—such as the ability to evade immune detection, secrete cell wall-degrading enzymes, or engage with plant signaling pathways—are capable of successful endophytic colonization [36]. Consequently, the root-inhabiting fungal community tends to exhibit reduced taxonomic complexity and is often dominated by a few specialized lineages, reflecting a “survival of the fittest few” assembly pattern [37]. This spatial gradient and selective filtering mechanism, first documented in coniferous forest systems such as Chinese fir, is broadly observed across a wide range of plant species, including key crops such as rice [38]. Accumulating evidence delineates a spatially structured continuum in plant–fungus interactions, characterized by a progressive transition from broad-spectrum microbial recruitment in the rhizosphere to stringent selective processes within the root endosphere [39]. This pattern reflects a pronounced ecological stability and evolutionary conservation among diverse plant lineages.

### 4.2. Rhizosphere Soil Is a Critical Fungal Reservoir for Root Colonization

The present study reveals that dominant fungal genera inhabiting root tissues, including *Russula*, *Tomentella*, *Subulicystidium*, and *Scytinostroma*, are also highly enriched in the surrounding rhizosphere soil. This pattern indicates that the rhizosphere functions as a principal inoculum reservoir for root-associated fungal communities. These findings align with the widely accepted view of the rhizosphere as both a microbial “seed bank” and a selective ecological filter. Ling et al. (2022) have emphasized this dual role in their comprehensive investigation of rhizosphere bacteriomes across diverse plant species and soil compartments [40]. Their work underscores that the rhizosphere not only harbors substantial microbial diversity but also facilitates selective microbial recruitment—a mechanism that transcends plant roots and fungi and applies broadly to bacterial communities and bulk soil environments.

Plant roots do not establish their microbiomes in isolation; rather, microbial communities are selectively recruited and enriched from the soil within the rhizosphere, a “microbial interface zone”, through the combined influence of root exudates, signaling molecules, and physical root architecture [41]. This selective assembly process from the inoculum bank is modulated not only by structural differences in root tissues, such as variations in permeability and nutrient exchange capacity between fine and thick roots, but also by the host’s genetic background, i.e., genotype characteristics. This study further revealed that certain fungal taxa colonize exclusively specific root tissues (e.g., *Delicatula* and *Scytalidium* were detected only in fine roots and thick roots) while remaining undetected in the rhizosphere soil. This suggests that root tissues function not merely as a passive “receiver” in fungal colonization but actively mediate selective recruitment and enrichment. Such selective colonization is likely influenced by tissue-specific microenvironments, including oxygen availability, pH, moisture content, and degree of lignification. Additionally, host genotype modulates root exudate composition and immune recognition mechanisms, thereby shaping the personalized assembly of root-associated microbiomes through differential recruitment and exclusion of specific microbial taxa.

Thus, rhizosphere soil, serving as the primary fungal reservoir, provides a critical source of microbial taxa for the plant root microbiome, while the ultimate colonization and symbiotic assemblage are jointly determined by host genotype and tissue-specific traits. This dual mechanism of exogenous input and host-mediated selection forms the ecological foundation of fungal community assembly in Chinese fir roots. It offers novel insights into plant–microbe interaction networks and provides a theoretical framework for the targeted manipulation of rhizosphere microbiomes, such as through the application of designed probiotic inoculants. Based on our findings, inoculants should be tailored to specific host genotypes (e.g., Russula for N96 and N103, Scytinostroma for N98 and N104) and applied to corresponding compartments (e.g., fine or thick roots) to maximize colonization efficiency. Such precision application may be achieved via root-zone inoculation during seedling establishment or soil amendment in early plantation stages.

### 4.3. Tissue and Host-Genotype Specificity in Structure and Function of Fungal Community

The present study revealed distinct spatial distributions of fungal communities across Chinese fir root compartments and rhizosphere soils, with *Russula* and *Tomentella* dominating in the rhizosphere, *Scytinostroma* and *Tomentella* enriched in fine roots, and *Subulicystidium* and *Scytinostroma* predominating in thick roots. Notably, *Delicatula* and *Scytalidium* were exclusively detected in root tissues, demonstrating a high degree of tissue specificity. These findings indicate that fungal taxa undergo strong compartmental filtering, which reflects both niche differentiation and functional adaptation to localized microenvironments. Additionally, certain host genotypes exhibited unique and consistent associations with specific fungal taxa across different compartments. For instance, *Russula* was highly enriched in the rhizosphere of N96 and N103, while *Tomentella* showed dominant enrichment in N94 and N97. In fine roots, *Scytinostroma* was particularly abundant in N98 and N104, and *Tomentella* in N97 and N102. *Russula* was also dominant in the fine roots of N103, accompanied by overall low diversity. Meanwhile, *Delicatula* was notably enriched in N104 and N94, indicating a possible affinity for those genotypes. In thick roots, *Subulicystidium* appeared broadly distributed but reached higher relative abundance in N94, N98, and N100, while *Scytinostroma* was especially enriched in N98. Other genotype–fungus associations included *Neonothopahus* dominance in N103, *Delicatula* enrichment in N95, and *Tomentella* dominance in N97 thick roots. These patterns underscore the pivotal role of host genotype in shaping root-associated fungal communities, likely mediated by differences in root exudation, immune responses, and tissue chemical composition.

The rhizosphere, characterized by abundant organic substrates and complex physicochemical gradients, acts as both a microbial “inoculum reservoir” and a selective ecological filter. Our results align with previous studies suggesting that rhizosphere microbial assembly is driven by root exudates, architecture, and genotype-mediated selection [40]. This dual function of the rhizosphere, previously established for bacteria and bulk soil, clearly extends to root-associated fungal communities. Moreover, our findings support the growing consensus that microbial selection is not random but tightly regulated by root tissue structure (e.g., permeability, lignification) and genotype-specific traits.

In woody plants such as Chinese fir, the extended lifespan of root systems enhances prolonged microbial interactions and selective pressure from the host, contributing to the establishment of a stable and functionally specialized microbiome [42]. Compared to herbaceous species, woody hosts display deeper temporal and spatial structuring of their microbiomes. Consistently, we found that certain fungi, traditionally classified as saprotrophs (e.g., Scytinostroma, Subulicystidium), were enriched in thick roots, likely due to their metabolic affinity for recalcitrant substrates such as lignin and hemicellulose. Such fungi may contribute to carbon turnover, secondary metabolism regulation, or pathogen suppression, thereby performing ecological functions beyond decomposition [43,44].

However, it is important to note that microbiomes are not exclusively shaped by host genotype. Studies on herbaceous species, including potato, have shown that genotype significantly influences microbiome composition and function [45]. Yet, environmental variables, agronomic management, and seasonality can exert equal or even greater influence than genotype, particularly in field conditions [46]. Although our study did not explicitly control for these external variables, their impact on microbial community structure must be acknowledged. In line with recent syntheses [47], we recognize that plant–microbiome assembly results from a complex interplay between endogenous factors (e.g., genotype, root traits) and exogenous drivers (e.g., soil type, climate, land-use history). Thus, our findings suggest that fungal community assembly in Chinese fir roots is shaped by a dual mechanism: inoculum supply from the rhizosphere and selective filtering by root compartments and host genotype. This framework provides mechanistic insight into root–microbiome interactions and establishes a foundation for targeted manipulation strategies, such as microbiome-based breeding or probiotic inoculant design. Future research should expand to include other compartments, such as bulk soil and the rhizoplane, and incorporate environmental variables to better resolve the relative contributions of host and habitat in shaping the root microbiome.

### 4.4. Key Fungal Genera Influencing Chinese Fir Growth

At the genus level, *Scytinostroma* exhibited significant positive correlations with all three growth parameters of *Cunninghamia lanceolata*, and its dominant OTU (OTU000004, *Scytinostroma ochroleucum*) showed consistent positive associations at the OTU level. These results suggest that *Scytinostroma* may play a critical role in promoting host plant growth. Previous studies have demonstrated that members of this genus possess selective lignin-degrading capabilities, preferentially decomposing lignin while preserving cellulose and hemicellulose [48]. As lignin is a major yet highly recalcitrant component of soil organic matter, its slow breakdown constrains carbon and nutrient turnover [49]. The selective delignification by *Scytinostroma* may enhance the mineralization efficiency of organic matter in the rhizosphere, thereby releasing nitrogen, phosphorus, and other essential nutrients that are otherwise sequestered within lignin matrices, ultimately improving nutrient bioavailability [50]. Moreover, this decomposition process likely facilitates the transformation of organic matter into stable humic substances, which improves soil structure, expands root growth space, and enhances microbial colonization potential. In addition, phenolic intermediates released during partial lignin degradation may participate in the regulation of root development, oxidative stress responses, and microbial signaling [51]. Thus, *Scytinostroma* may not only promote host growth by contributing to nutrient cycling but also by reshaping the rhizosphere microenvironment and signaling networks, thereby enhancing plant adaptability to environmental conditions. However, despite its apparent functional potential, the ecological roles and growth-promoting mechanisms of *Scytinostroma* remain largely unexplored and warrant further investigation.

Fungi belonging to the genera *Neonothopanus* and *Lachnum* exhibited significant negative correlations with the height of *Cunninghamia lanceolata*, suggesting that their colonization in the root or rhizosphere environment may impose inhibitory effects on host plant growth. The genus *Neonothopanus* is a representative bioluminescent fungal taxon whose luminescence arises from the oxidation of luciferin catalyzed by luciferase [52]. This reaction is accompanied by the continuous generation of reactive oxygen species (ROS). At low levels, ROS function as signaling molecules involved in the regulation of plant–microbe interactions. However, their excessive accumulation can induce lipid peroxidation in root cell membranes and disrupt antioxidant homeostasis, thereby impairing root functionality and compromising water and nutrient uptake [53]. Moreover, luciferin and its metabolic byproducts are not intrinsic components of the plant signaling network. Their potential bioluminescent activity in the rhizosphere may interfere with plant photoreception, disrupt root phototropic or chemotropic responses, and consequently restrict spatial root expansion, ultimately leading to suppressed plant growth. Additionally, the colonization and bioluminescence of *Neonothopanus* depend on carbon sources exuded by the host roots to sustain hyphal growth and metabolic activity. This microbial demand for carbon may perturb the host’s carbon allocation and energy regulation, thereby further diminishing its growth potential.

Recent studies have shown that fungi of the genus *Neonothopanus* are capable of producing a variety of bioactive secondary metabolites, including alkaloids and tannin-like compounds [54]. Alkaloids can mimic or interfere with plant hormone signals, affecting the polar transport and differentiation of root tip cells, thereby inhibiting root elongation and branching. Tannins inhibit cell expansion by binding to cell wall proteins and simultaneously chelate mineral ions such as Fe^2+^, Mg^2+^, and Ca^2+^, reducing their availability and thus affecting nutrient uptake. Current research on fungi of the genus *Neonothopanus* primarily focuses on their bioluminescent properties, while their potential ecological functions and negative regulatory effects on host plant growth warrant further in-depth investigation.

*Lachnum* is a widely distributed genus of saprophytic fungi within the fungal kingdom [55], known to biosynthesize a diverse array of bioactive secondary metabolites. These metabolites include compounds exhibiting antimicrobial, nematicidal, and cytotoxic activities, such as lachnumon, lachnumol A, and mycorrhizin A, which have been isolated from *Lachnum papyraceum* [56]. These compounds inhibit various bacteria and fungi, indicating their potential role in shaping the structure and function of the rhizosphere microbial community. As part of the rhizosphere microbiome, some fungi may directly suppress root pathogens by competing for ecological niches or secreting antimicrobial substances. This activity contributes to the microbial barrier within the plant defense system. Additionally, mycorrhizal fungi and other microbes can induce systemic resistance in plants, enhancing their defense against root pathogens [57]. Given that the growth of Cunninghamia lanceolata relies heavily on beneficial rhizosphere microorganisms, especially arbuscular mycorrhizal fungi and nitrogen-fixing bacteria, the specific effects of secondary metabolites from *Lachnum* species on these symbionts require further investigation.

It is noteworthy that there are interspecific differences in disease susceptibility and impacts among different tree species [58]. Our study found that different genotypes of the same tree species also have differences in the fungal communities colonizing various parts. The phenotype of the host can shape the structure of its microbiome, allowing different plant genotypes to colonize different microbial communities [59]. Similar phenomena have been confirmed in poplar studies: the structure of root EMF communities depends on the genotypic characteristics of their hosts [60]. In addition, the low abundance distribution of plant pathogens in all tissues in this study confirms the inhibitory effect of rhizosphere-beneficial microorganisms (such as AMFs) on pathogens. Studies have shown that AMFs can enhance plants’ resistance to biological stress by inducing systemic resistance [61].

## Data Availability

ITS amplicon relative sequencing raw sequences were deposited in NCBI with accession number PRJNA1268739.

## Abbreviations

The following abbreviations are used in this manuscript:

NMDS	Non-metric multidimensional scaling
FRs	fine roots
RS	rhizosphere soil
TRs	thick roots
OTUs	Operational Taxonomic Units
AMFs	arbuscular mycorrhizal fungi
PRRs	pattern recognition receptors
ISR	inducing systemic resistance
ECMs	ectomycorrhizal fungi
